# The Delivery of α1-Antitrypsin Therapy Through Transepidermal Route: Worthwhile to Explore

**DOI:** 10.3389/fphar.2020.00983

**Published:** 2020-07-03

**Authors:** Srinu Tumpara, Beatriz Martinez-Delgado, Gema Gomez-Mariano, Bin Liu, David S. DeLuca, Elena Korenbaum, Danny Jonigk, Frank Jugert, Florian M. Wurm, Maria J. Wurm, Tobias Welte, Sabina Janciauskiene

**Affiliations:** ^1^ Department of Internal Medicine, Biomedical Research in Endstage and Obstructive Lung Disease Hannover (BREATH), Member of the German Center for Lung Research (DZL), Hannover, Germany; ^2^ Molecular Genetics Unit, Instituto de Investigación de Enfermedades Raras (IIER), Instituto de Salud Carlos III (ISCIII), Madrid, Spain; ^3^ Research Core Unit for Structural Biochemistry, Hannover Medical School, Hannover, Germany; ^4^ Institute of Pathology, Hannover Medical School, Hannover, Germany; ^5^ Department of Dermatology, University Clinic Aachen, Aachen, Germany; ^6^ ExcellGene SA, Monthey, Switzerland; ^7^ Faculty of Life Sciences, Ecole Polytechnique Federale de Lausanne, Lausanne, Switzerland

**Keywords:** alpha1-antitrypin, Inflammation Immunomodulation, epidermis, topical-Skin cream, RNA-Seq-RNA sequencing

## Abstract

Human α1-antitrypsin (AAT) is an abundant acute phase glycoprotein expressing anti-protease and immunomodulatory activities, and is used as a biopharmaceutical to treat patients with inherited AAT deficiency. The pleiotropic properties of AAT provide a rationale for using this therapy outside of inherited AAT deficiency. Therapy with AAT is administrated intravenously, yet the alternative routes are being considered. To examine the putative transepidermal application of AAT we used epiCS®, the 3D human epidermis equivalents reconstructed from human primary epidermal keratinocytes. We topically applied various concentrations of AAT protein with a constant volume of 50 µl, prepared in Hank’s balance solution, HBSS, to epiCS cultured under bas\al condition or when culture medium supplemented with 100 µg/ml of a combined bacterial lipopolysaccharide (LPS) and peptidoglycan (PGN) mixture. AAT freely diffused across epidermis layers in a concentration and time-dependent manner. Within 18 h topically provided 0.2 mg AAT penetrated well the stratum corneum and localizes within the keratinocytes. The treatments with AAT did not induce obvious morphological changes and damages in keratinocyte layers. As expected, LPS/PGN triggered a strong pro-inflammatory activation of epiCS. AAT exhibited a limited capacity to neutralize the effect of LPS/PGN, but more importantly, it lowered expression of IL-18 and IL-8, and preserved levels of filaggrin, a key protein for maintaining the epidermal barrier integrity. Our findings suggest that the transepidermal route for delivering AAT is worthwhile to explore further. If successful, this approach may offer an easy-to-use therapy with AAT for skin inflammatory diseases.

## Introduction

Human alpha1-antitrypsin (AAT) encoded by SERPINA1 gene is an acute phase glycoprotein, a member of the serpin (serine protease inhibitor) superfamily. Under healthy conditions, plasma levels of AAT range between 1.3 and 2 g/L, whereas during acute inflammation or infection these levels increase four to five times of the normal range. This protein plays an important role in preventing the proteolysis amplification during acute inflammation and organ injury (Janciauskiene et al., 2011). Currently, intravenous infusions of human plasma-purified AAT preparations are used for the treatment of lung diseases associated with severe inherited AAT deficiency (circulating levels of AAT about 10% of normal). Inherited AAT deficiency can also be associated with other inflammatory disorders, such as antiproteinase-3-associated vasculitis, panniculitis, psoriasis, chronic urticarial, etc. (Blanco et al., 2017; Torres-Duran et al., 2018). Results from the single cases and small patient cohorts show that AAT therapy is very effective in AAT deficient patients with panniculitis (Sandhaus et al., 2016; Miravitlles et al., 2017).

The anti-apoptotic and immunomodulatory properties of AAT provide a rationale for testing AAT preparations outside of inherited AAT deficiency. According to the results from various experimental models, therapy with AAT is beneficial in preventing transplant rejection, ischemia-reperfusion injury, graft-versus-host disease, experimental autoimmune encephalomyelitis, preeclampsia, and inflamed pancreatic islets, among others (Lewis et al., 2005; Lewis et al., 2008; Grimstein et al., 2010; Gao et al., 2014). Few clinical trials have been conducted to address the potential benefit of AAT therapy for conditions unrelated to inherited AAT deficiency, such as islet and lung transplantation, type 1 diabetes, graft-versus-host disease, acute myocardial infarction, and cystic fibrosis (Gottlieb et al., 2014; Sorrells et al., 2015; Rachmiel et al., 2016; Brener et al., 2018).

The promising broad-spectrum therapeutic effects of AAT encourage scientists to seek for the novel administration routes of AAT biopharmaceuticals. For example, currently inhaled AAT is investigated for its biochemical efficacy and safety in patients with lung diseases (Stolk et al., 2019). A subcutaneous administration of AAT therapy is being explored as well (Cowden et al., 2005). Historically, few pilot and feasibility studies investigated transdermal delivery of AAT as well. Wachter and co-authors have shown a significant clinical improvement in six patients with atopic dermatitis who used creams containing 1–5% of AAT. Authors reported that within 6 to 21 days pain and pruritus disappeared, and tissue healing increased in all six patients. No adverse side effect of such therapy was documented (Wachter and Lezdey, 1992). This study provided the first evidence that topical application of AAT is possible. On the other hand, when recombinant AAT has been administered topically for 3 weeks to five patients with Netherton syndrome (a disorder that affects the skin, hair, and immune system) no beneficial effect was observed (Mazereeuw-Hautier et al., 2006). It remained unclear whether this was due to a formulation issue or the property of the recombinant AAT *per se*. Although results from these two pioneering studies are inconclusive, topical application of acute phase proteins like AAT appears a promising avenue for future research (Jung and Stingl, 2008). To date, no studies have been conducted to evaluate the concentration and time-dependent effects of AAT on human epidermis, and the question if AAT can be useful for transepidermal applications remains not answered.

According to previous studies, AAT enters the tissues from the circulation by passive diffusion *via* cell membranes (Stockley, 1984). For example, neutrophils, peripheral blood mononuclear cells, cancer cells, and endothelial cells cultured in the medium supplemented with AAT all show cytoplasmic immunostaining for AAT (Sohrab et al., 2009; Janciauskiene et al., 2011; Jonigk et al., 2013; Ercetin et al., 2019). In line with this, experimental studies revealed that AAT added to the apical or the basolateral side of the endothelium appears intracellularly and secreted across the trans-well membrane, suggesting bidirectional transport of AAT across the endothelium (Ferkol et al., 2000; Vogel and Larsen, 2000; Lockett, 2017). It is also important to point out that AAT interacts with a wide range of hydrophobic ligands including plasma lipoproteins (Gordon et al., 2015), cell membrane lipid rafts (Subramaniyam et al., 2010), cholesterol (Janciauskiene and Welte, 2016), fatty acids (Frenzel et al., 2015), and free heme (Janciauskiene et al., 2017). These latter properties of AAT might be essential for epidermal barrier permeability and hence encourage further validation of AAT protein as a putative candidate for developing topical therapeutics.

In this study, we used the 3D human epiCS model reconstructed from normal human primary epidermal keratinocytes, which resemble human epidermis containing a basement membrane, proliferating keratinocytes, and a stratum corneum with an intact barrier function. Our aim was to study free diffusion of AAT across epidermis. We asked: i) can topically applied AAT penetrate epiCS; ii) if yes, does AAT cause physical and chemical alterations in epiCS, and iii) if epiCS are strongly stimulated with a mixture of bacterial lipopolysaccharide (LPS) and peptidoglycan (PGN), can topically applied AAT neutralize some of the pro-inflammatory effects of these stimuli?

## Materials and Methods

### EpiCS

EpiCS®, three dimensional (3D), fully differentiated human epidermis equivalent, reconstructed from normal human epidermal keratinocytes were purchased from CellSystems® (Troisdorf, Germany). The cellular structure of reconstructed human epidermis closely resembles the human epidermis including proliferating keratinocytes and a stratum corneum with intact barrier function. EpiCS® is an OECD certified model for the testing of chemicals (Section 4, TG 431 and 439) (www.oecd.org). For our experiments, we purchased 13 separate batches of epiCs in total. Each epiCS batch was tested for contaminants from primary cells (HIV, HBV, and HCV), sterility (bacteria, yeast, and mycoplasma), and validated histologically as described by (Hoffmann et al., 2005). EpiCS were cultured in a special culture medium (Cat. Nr. CS-3051, CellSystems®).

### Alpha1-Antitrypsin (AAT)

Plasma purified human AAT (99% purity, Zemaira, CSL Behring, Kankakee, IL, USA) was used for experiments after buffer exchange to the sterile Hank’s balance solution (L2045, Merck Millipore-Germany), by using 10K centrifugal filter columns (Thermo Fischer #88517). The protein concentration was determined using the BCA Protein Assay Kit (Pierce™, Rockford, IL) according to instructions of the supplier. In some confirmatory experiments, we used a highly purified (83%), glycosylated form of recombinant AAT protein produced in Chinese hamster ovary (CHO) cells (gift from ExcellGene, Monthey, Switzerland). The quality of both proteins is shown in 10% SDS-PAGE.

### Experimental Conditions

EpiCS were treated with various concentrations of AAT added topically (always in a constant volume of 50 µl) or into the culture medium (basolaterally) for different time points up to 48 h. The control epiCS were treated by adding the same volume of Hank’s balance salt solution (HBSS cat nr. 14025092, ThermoFisher Scientific, Germany) as for the AAT treatments. Additionally, epiCS were cultured for 18 h in the medium containing 100 µg/ml of combined bacterial lipopolysaccharide (LPS, Sigma-Aldrich, Darmstadt, Germany) and peptidoglycan (PGN, stock solution prepared in 0.1 mg/ml in sterile water and few seconds sonicated; Sigma-Aldrich) mixture with and without topical application of 0.2 mg of native or recombinant AAT. At the end of the experiment, epiCs culture medium and cells were collected for the further studies.

### Immunohistochemistry

We fixed epiCS with 4% neutral-buffered formalin, dehydrated and embedded it in formalin-fixed paraffin-embedded (FFPE) blocks. A rotary microtome was used to prepare the five-micron thick sections that were mounted on the slides. Afterwards, tissue sections were deparaffinized and antigen retrieval was achieved by heat-induced epitope retrieval (HIER) method based on 10 mM citric acid buffer (pH 6.0). Endogenous peroxidases were blocked with 3% H_2_O_2_ for 10 min at RT and unspecific antigens were blocked with 10% fetal calf serum (FCS) in phosphate buffered saline (PBS, pH 7.4) for 1h at RT. Afterwards, sections were incubated overnight with primary antibodies: rabbit polyclonal anti-human AAT (1:5,000, A0012 DAKO, Copenhagen, Denmark), mouse monoclonal IgG1 (kappa light chain) anti-filaggrin (1:500, AKH1, sc66192c, Santa Cruz Biotechnology, Inc. Heidelberg Germany), or rabbit polyclonal anti-SREBP1 antibody (ab28481, Abcam, Cambridge, United Kingdom). Afterwards, slides were washed and mounted on a coverslips using a drop of ProLong™ Gold Antifade Mountant containing DAPI (Thermo Fisher scientific Waltham, Massachusetts, USA). Images were taken at 20× or 40× magnifications with the iRiSTM Digital Cell Imaging multicolor fluorescence system (Logos Biosystems,Villeneuve d’Ascq, France).

For some experiments, slides were incubated for 1 h at RT with fluorescent-tagged secondary antibodies (Goat anti-Rabbit IgG [H+L] Highly Cross-Adsorbed Secondary Antibody, Alexa Fluor 488 from Thermo Fisher Scientific, catalog #A-11034 or Goat anti-Mouse IgG [H+L] Cross-Adsorbed Secondary Antibody, Alexa Fluor 488 from Thermo Fisher Scientific, #A-11001). When sections were washed with PBS and mounted using DAPI containing medium (counterstained with 4′-6-diamidino-2-phenylindole, Thermo Fisher Scientific). Images were taken with an Olympus FluorView 1000 scanning confocal microscope equipped with a 60× oil immersion objective.

We also employed a more sensitive chromogenic protein detection system using DAB (3,3′-diaminobenzidine tetrahydrochloride). Endogenous peroxidases were blocked with 3% H_2_O_2_ for 10 min at RT and unspecific antigens were blocked with 10% FCS in PBS, pH 7.4, for 1h at RT. Tissue sections were incubated with rabbit polyclonal anti-human AAT antibody (A0012- DAKO, Denmark) at 40°C overnight. Thereafter, sections were incubated with HRP polymer (GTX-83398- Gene Tex), for 45 min and with DAB (DAB substrate kit, Zytomed, Germany) for 15 min at RT. Slides were then counterstained with hematoxylin, mounted with DPX (dibutyl phthalate xylene), and analyzed under a light microscope. Images were taken at 40× or 100× magnification using Leica DM750 microscope equipped with Leica ICC50 HD camera (Leica Microsystems, Wetzlar, Germany).

### Western Blots

By the end of an experiment, epiCS culture medium was collected and epiCs cells were lysed in RIPA buffer (20 mM Tris-HCl pH 7.5, 150 mM NaCl, 9.5 mM EDTA, 1% Triton X-100, 0.1% SDS, and 1% sodium deoxy-cholate) (R0278, Sigma-Aldrich), supplemented with protease inhibitor cocktail (P8340, Sigma-Aldrich). Protein concentrations were determined using the bicinchoninic acid (BCA) kit (23227-Thermofischer Scientific, USA), according to the manufacturer’s instructions. The protein contents were measured using a Tecan Infinite 200 PRO (Männedorf, Switzerland). Equal volumes of epiCs culture medium or equal amounts of lysed proteins were separated by 10% SDS-polyacrylamide gels prior to transfer onto a polyvinylidene difluoride (PVDF) membranes (Millipore, Billerica, MA, USA). Membranes were blocked for 1 h with TBS+0.01% tween containing 5% low fat milk powder (Roth, Karlsruhe, Germany) followed by overnight incubation at 4°C with primary antibody polyclonal rabbit anti-human AAT (1:800, catalog # A0012 -DAKO, Denmark), mouse monoclonal anti-vimentin antibody (1:500, catalog# WH0007431M1, Sigma), mouse monoclonal anti-cytokeratin 4 (1:500, catalog # AB9004, Abcam), and rabbit monoclonal anti-cytokeratin 13 (1:500, catalog # AB239918, Abcam). The immune complexes were visualized with horseradish peroxidase-conjugated antibody (P0217-DAKO, Denmark) and enhanced by ECL western blotting substrate (catalog #170-5060-BIO-RAD). Images were taken by using Chemidoc Touch imaging system (BioRad, Hercules, CA, USA).

### ELISA

Cell culture medium was briefly centrifuged and analyzed directly or stored at −80°C. ELISA kits for IL-8 (DY208), assay detection range (31.3–2,000 pg/ml), IL-18 (DY318-05, assay detection range 11.7–750 pg/ml), TNF-α (DY210, assay detection range 15.6–1,000 pg/ml), IL-6 (DY206-05, assay detection range 9.38–600 pg/ml), and IL-1 α (DY200-05, assay detection range 7.81–500 pg/ml), were purchased from (R&D Systems Inc., Minneapolis, MN, USA) and used according to the manufacturer’s instructions. Plates were analyzed by microplate reader (Tecan Infinite M200) measuring absorbance at 450 nm with the correction wavelength set at 540 or 570 nm.

### RNA Sequencing (RNA-Seq)

The total RNA from epiCS cells was isolated, and its quality was assessed using 1% agarose gels as well as by Agilent 2100 Bioanalyzer using Agilent RNA 6000 Nano Kit. Libraries were prepared in duplicates by using 200 ng RNA of each insert. TruSeq Stranded mRNA Kit (Illumina Inc., USA) was used for library preparation according to the manufacturer protocol as described in our previous work (Ercetin et al., 2019). The products were purified with Ampure XP Beads (Beckman Coulter, CA, USA) and enriched with 15 cycles of PCR to create the cDNA library. Sequencing was performed at the Genomics service (ISCIII) on a NextSeq 500 sequencer using 75 base read lengths in paired-end mode. The obtained RNA-Seq data was analyzed by the Bioinformatics Facility (ISCIII). A quality control analysis was based on fastQC v0.11.3 (http://www.bioinformatics.babraham.ac.uk/projects/fastqc/) and any adapter sequences and low quality 3′ ends were removed using Trimmomatic v0.36. High-quality reads were mapped against Hg38 human genome using STAR v2.4.0.1 and mapping quality control was performed using RseQC v2.6.4. Transcriptome prediction and gene/isoform quantification was calculated using HT-seq v0.11.1 based on Hg38 RefSeq reference genes. RNA-seq data accession number is following: GSE150946 (https://www.ncbi.nlm.nih.gov/geo/query/acc.cgi?acc=GSE150946).

### Analysis of RNA-Seq Data

Original RNA sequencing data in FASTQ form was aligned by STAR v2.4.0.1 (Dobin et al., 2013). The mapped reads were counted by HT-seq v0.11.1 to quantify gene expression levels (Anders et al., 2015). The differential expression (DE) was subsequently quantified using generalized fold change (GFOLD) scores with GFOLD v1.1.4 P (Feng et al., 2012). ANTHER Classification System was used to determine significantly enriched GO terms (Mi et al., 2019).

### RT-PCR

Total RNA was prepared using the RNeasy Mini kit (Qiagen Sample and Assay Technologies; Qiagen, Valencia, CA, USA.). The RNA amounts were determined with the NanoDrop spectrophotometer (Thermo Scientific, Bremen, Germany). For cDNA synthesis, 1 µg total RNA was transcribed using a High Capacity cDNA Reverse Transcription Kit (Thermo Fisher Scientific). Levels of mRNA of selected genes (**Table 1**) were analyzed using TaqMan Gene Expression Assays (Thermo Fisher Scientific) on a StepOnePlus Real‐Time PCR Systems machine (Thermo Fisher Scientific). All taqman primers we purchased from Thermo Fisher Scientific. The Ct value for each sample was calculated by determining the point at which the fluorescence exceeded a threshold limit. HPRT was used as a housekeeping gene in the same run. Basal expression of genes was calculated according to the method 2ΔCt (Ct value of target gene − Ct value of reference gene). All measurements were performed in duplicates, with samples from three independent experiments.

**Table 1:** Taqman primers list used for RT- PCR.

Primer	Assay ID
*COX2*	Hs00153133_m1
*IL8 (CXCL8)*	Hs00174103_m1
*FLG2*	Hs00418578_m1
*HPRT1*	Hs02800695_m1
*IL-1α*	Hs00174092_m1
*IL-18*	Hs01038788_m1
*SREBF1*	Hs01088691_m1
*SOD2*	Hs00167309_m1
*TGFβ1*	Hs00998133_m1
*VIM*	Hs00958111_m1
*KRT4*	Hs00361611_m1
*KRT13*	Hs 00999762_m1
*COL7A1*	Hs00164310_m1

### Statistical Analysis

Data of qPCR, and ELISA analyses were statistically analyzed and presented using Sigma Plot 12.5 software package (Systat Software GmbH, Erkrathor, Germany). Student’s t-test was applied to compare two sample means on one variable. When more than two groups were involved in the comparison, one-way ANOVA was used. Data were presented as mean and standard deviation if normality test did not fail. A p-value of less than 0.05 was considered significant.

## Results

### Topically Applied AAT Has No Effect on Morphological Characteristics of EpiCS

**Figure 1:**
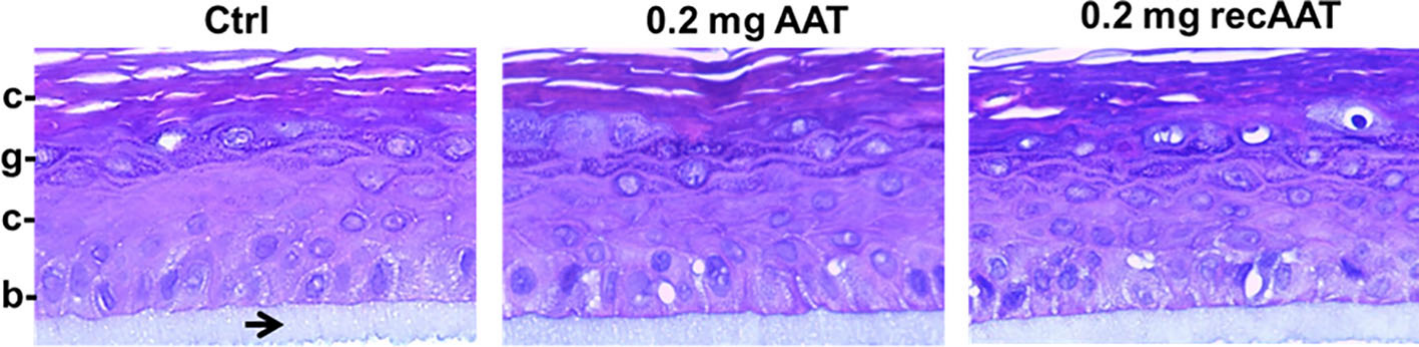
Typical images of the tissue architecture of the 3D reconstructed human epidermis culture incubated for 18 h without or with topical application of 0.2 mg human AAT or recombinant (recAAT). The epidermis model was formalin-fixed, embedded in paraffin, cut, and stained with hematoxylin/eosin. The inserts were prepared for histology while still adhering to the polycarbonate (PC) insert membrane (indicated by arrow). Images were taken at 40× magnifications with the iRiSTM Digital Cell Imaging multicolour fluorescence system. b, *stratum basale* (basal layer); s, *stratum spinosum* (spinous layer); g, *stratum granulosum* (granular layer); c, *stratum corneum* (horny layer, the outermost layer of the epidermis).

The 3D human epidermis equivalents (epiCS) were cultured for 18 h with or without topical application (onto the stratum corneum) of 50 µl native or recombinant AAT (0.2 mg). After that, we cut out layered epidermis from the inserts, fixed in 4% buffered formaldehyde solution, and embedded in paraffin. Sections were subsequently stained with hematoxylin/eosin solution. As illustrated in **Figure 1**, the epidermal model consisted of typical layers of keratinocytes, revealing the tissue-specific differentiation pattern. The topical application of 0.2 mg AAT proteins did not induce noticeable effect on the thickness or morphology of the epidermal cell layers. Similarly, no morphological differences we observed between controls (HBSS treated) and 0.5 mg AAT treated epiCS (**Supplementary Figure 1**).

### AAT Protein Freely Penetrates Keratinocyte Layers in a Concentration-Dependent Manner

**Figure 2:**
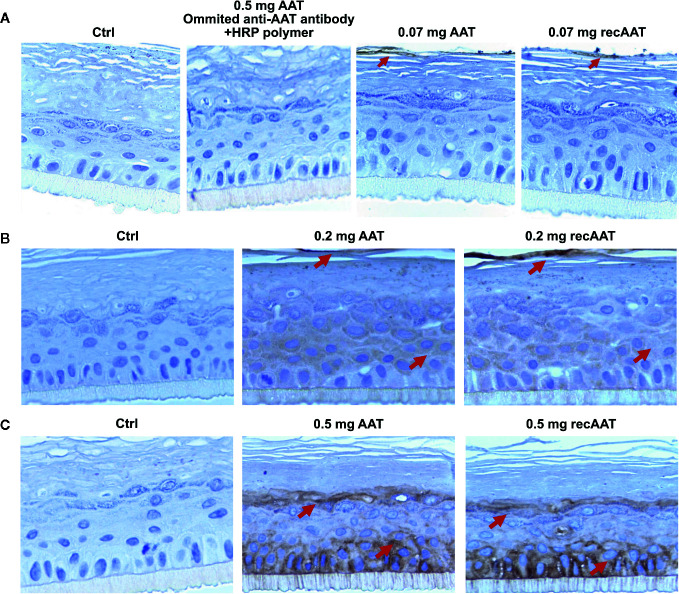
**(A–C)**. Human AAT and recombinant (recAAT) diffuse *via* epiCS in a concentration-dependent manner: **(A)** 0.07 mg; **(B)** 0.2 mg, and **(C)** 0.5 mg of AAT added topically for 18 h. After endogenous peroxidases were blocked with 3% peroxide, sections were incubated with rabbit polyclonal anti-human AAT antibody (1:5,000) and stained using an HRP-DAB (3,3′-diaminobenzidine tetrahydrochloride) as described in *Materials and Methods*. In a separate experiment, epiCS topically treated with 0.5 mg AAT were incubated with HRP polymer excluding primary anti-AAT antibody. This control eliminated the possibility that the HRP polymer non-specifically binds to AAT (A panel). Images are representative for 10 taken from different areas of each specimen. Staining for AAT (brown color) indicated by arrows. Images were taken at 100× magnifications using Leica DM750 microscope equipped with Leica ICC50 HD camera.

In the following experiments, we cultured epiCS for 18 h with topically applied equal volumes of 0.07, 0.2, or 0.5 mg of human AAT or recombinant (recAAT). As illustrated in **Figure 2A**, no positive staining for AAT was found in epiCS treated with HBSS (negative control) or in epiCS treated with 0.5 mg AAT and incubated with horseradish peroxidase (HRP) polymer without using primary anti-AAT antibody (procedural control). When we topically applied 0.07 mg AAT, only a weak AAT staining occurred in the outermost layer of the epidermis. By contract, specimens generated from the epiCS treated with 0.2 mg AAT and especially with 0.5 mg AAT, showed positive AAT staining in the outermost layer of the epidermis (*stratum corneum*) but also in the basal layer (*stratum basale*) and other layers of the epidermis. Remarkably, some of the cell areas were very strongly positive for AAT (**Figures 2B, C** **)** and **Supplementary Figures 2A, B**.

### Proteins Released Into the EpiCS Culture Supernatants in a Time-Dependent Manner

**Figure 3:**
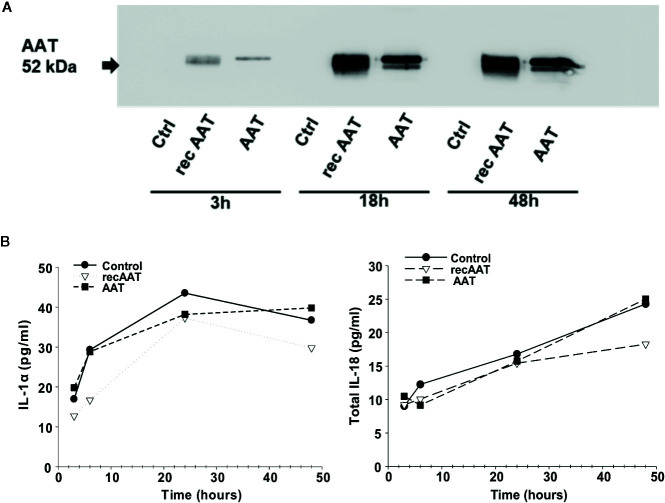
**(A**, **B)**. Topically added 0.2 mg AAT freely diffuses *via* epiCS in a time-dependent manner and is detected in epiCS culture supernatants **(A)**. Independently of AAT, epiCS increase IL-1α and total IL-18 release within 48 h of culture **(B)**. Each point in the graphs represents two independent repeats.

We next analyzed AAT protein in medium collected from epiCS cultures by Western blotting. For these experiments, we decided to add 0.2 mg of human or recombinant AAT topically and incubated epiCS for 3, 6, 18, and 48 h at 37°C, 0.5% CO_2_. The analysis of culture medium revealed that only after 3 h a small amount of AAT could be detected (**Figure 3A**). Similarly, very low levels of AAT were detected after 6 h (data not shown). However, marked increase in detectable AAT was seen after 18 and 48 h. Notably, supernatants collected from control epiCS (treated with HBSS) were negative for AAT during all incubation periods. Furthermore, over time of epiCS culture the secretion of IL-1α and total IL-18 in the media increased. However, topically applied 0.2 mg human AAT or recAAT did not influence basal levels of these cytokines (**Figure 3B**), implying that AAT does not induce damage or activation of epiCs. For the following experiments, the topical application of 0.2 mg AAT for 18 h was chosen as a standard experimental condition.

### AAT Applied to EpiCS Localizes in the Cell Cytosol

To examine the intracellular localization of AAT by confocal-laser scanning microscopy, we added 0.2 mg human AAT or recAAT on the top or into the culture medium (basolateral) of epiCS and incubated for 18 h. As shown in **Supplementary Figure 3A**, basolateral application of AAT resulted into the positive AAT staining throughout all keratinocyte layers except the outermost layer of the epidermis (*stratum corneum*). Remarkably, AAT protein was present in the cytosol (*green fluorescence*) but not in the nuclei of keratinocytes. Similar experiments performed using a topical application of 0.2 mg AAT showed very strong fluorescence staining on the outermost layer of the epidermis and only a slight staining within keratinocyte cytosol (**Supplementary Figure 3B**). One potential limitation of this approach, however, is the strong endogenous fluorescence of skin models (Hermsmeier et al., 2018).

### Topically Applied 0.2 mg of AAT Does Not Induce EpiCs Irritation as Determined by Cytokine Release

Other authors have shown that activated keratinocytes respond by secreting cytokines, such as IL-8, IL-18, IL-1α, and TNF-α (Gibbs et al., 2013; Galbiati et al., 2017). As illustrated in **Supplementary Figure 4**, neither of the two AAT protein preparations induced IL-8 and IL-18 release. By contract, AAT lowered IL-8 release relative to controls. Under basal conditions, epiCs secreted very low levels of IL-1α and IL-6. However, neither human AAT nor recAAT affected IL-1α release if compared to controls [(mean (SD), n = 3 independent experiments, AAT 68.5 (35) *vs* control 63.6 (23.8) pg/ml and recAAT 60.1 (25.9) *vs* control 63.6 (23.8) pg/ml), n.s.]. Similarly, when compared to controls, AAT had no effect on IL-6 levels [mean n = 3 independent experiments: control 0.26 (0.04) *vs* AAT 0.24 (0.05) and *vs* recAAT 0.24 (0.1) pg/ml, n.s.). We also measured TNF-α, however values were below the detection limits (data not shown).

### Effects of Topically Applied 0.2 mg AAT on Vimentin and Filaggrin Proteins

**Figure 4:**
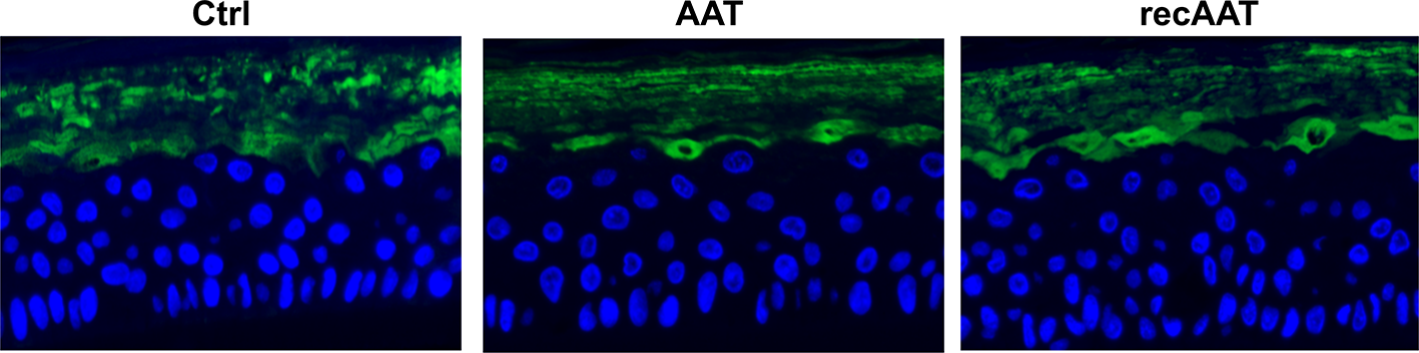
Immunofluorescence microscopy of filaggrin in the upper granular and cornfield layers of epiCS. The epiCS with topically applied 0.2 mg of human AAT or recAAT and were processed after 18 h as described in *Materials and Methods*. Sections were incubated overnight with mouse monoclonal IgG1 (kappa light chain) anti-filaggrin 1:500 (AKH1, sc66192c, Santa Cruz), washed, and mounted on a coverslip using a drop of ProLong™ Gold Antifade Mountant containing DAPI. Images were taken at 20× magnification with the iRiSTM Digital Cell Imaging multicolor fluorescence system (Logos Biosystems, France). Representative images show filaggrin (*in green*) and cell nucleus (*in blue*). Scale bars: 20 µm (not shown).

**Figure 5:**
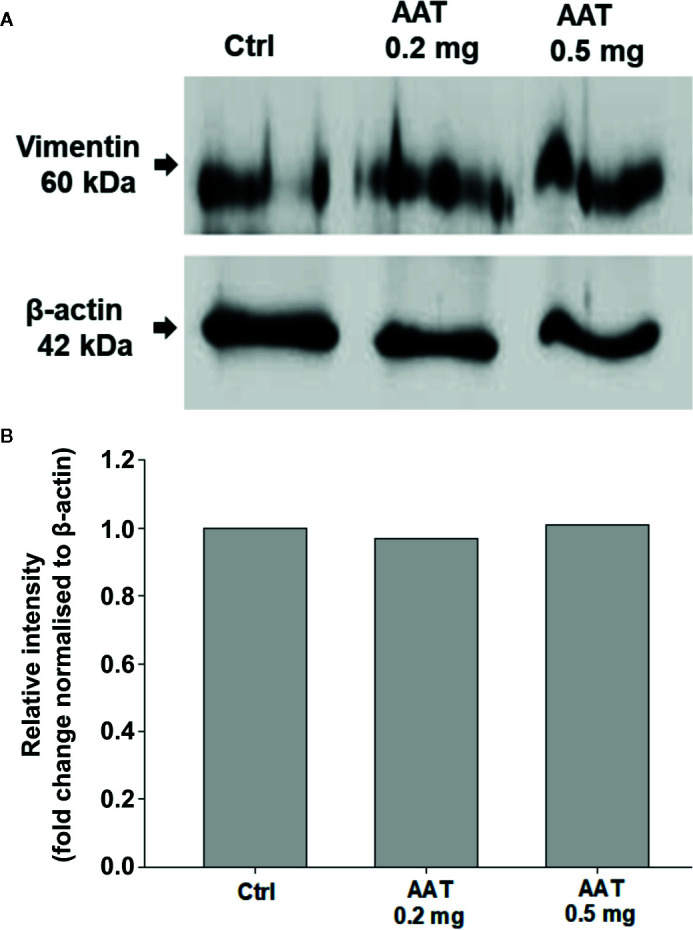
Effects of topically applied human AAT on vimentin levels. EpiCS were topically treated with 0.2 and 0.5 mg AAT for 18 h, lysed in radioimmunoprecipitation assay buffer (RIPA, Sigma), and analyzed by Western blotting using mouse monoclonal anti-vimentin antibody (1:500, Sigma). Both concentrations of AAT had no effect on vimentin protein levels. Representative blot is shown **(A)**. Bar charts display relative intensity of vimentin bands from two independent experiments **(B)**.

Filaggrin (FLG, filament-aggregating protein) is a major component of the protein-lipid envelope of the epidermis and important for water permeability and the entry of microbes and allergens (Asselineau et al., 1990). According to the immunohistochemistry results, topically applied 0.2 mg AAT or recAAT had no significant effect on FLG protein levels relative to controls (**Figure 4**). Likewise, levels of vimentin, a type III intermediate filament that regulates would healing, remained unaffected by the treatments with 0.2 or 0.5 mg AAT (**Figure 5**). Furthermore, AAT had no effect on vimentin (*VIM*) gene expression: [mean *VIM* mRNA relative to housekeeping gene HPRT, n = 3 independent experiments, 6 repeats: 0.2 mg AAT 19.1 (1.1) *vs* controls 18.9 (0.9), n.s.]. By contrast, topically applied 0.2 mg AAT reduced *FLG2* gene expression: [*FLG2* mRNA relative to housekeeping gene HPRT1, mean n = 3 independent experiments, 6 repeats: AAT 0.41 (0.03) *vs* controls 1.29 (0.04), p < 0.001].

### The Expression of *COX2*, *TGFB1*, *SOD2*, and *SREBF1* Genes in EpiCS Treated With AAT

**Figure 6:**
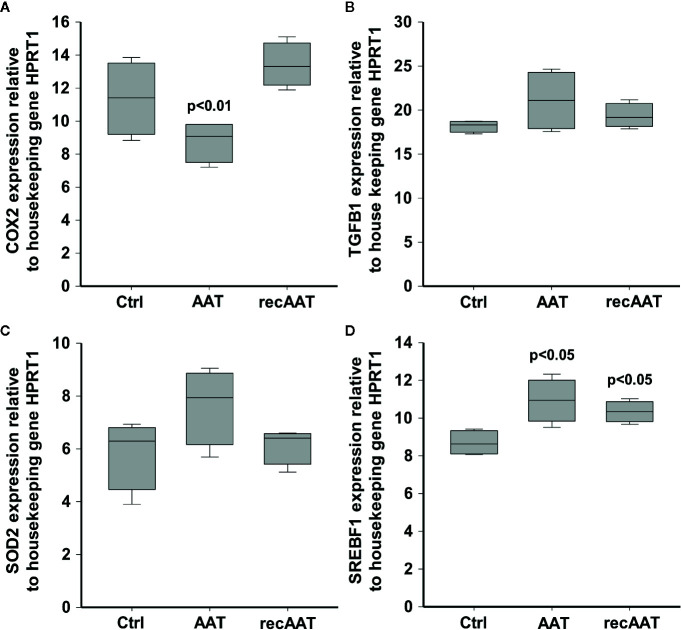
Effects of topically applied 0.2 mg human AAT and recAAT on gene expression. The expression of *COX2* **(A)**, *TGFB1* **(B)**, *SOD2* **(C)**, and *SREBF1* **(D)** genes was determined by real-time PCR as described in *Materials and Methods*. HPRT1 was used as a housekeeping gene. Box plots represent mean from three individual epiCS with two replicates for each experiment. P value indicates significant differences compared to the values seen in controls.

The results above prompted us to investigate an expression of a few specific genes by RT-PCR. The mRNA was prepared from epiCS topically treated with 0.2 mg AAT or recAAT for 18 h. COX2, a highly inducible gene in response to a variety of pro-inflammatory agents and cytokines (Wu, 1996), was not affected by recAAT but significantly lowered by human AAT (**Figure 6**). Neither of AAT proteins showed an effect on the expression of transforming growth factor (TGF)-β1, a mediator of cell proliferation, differentiation, and extracellular matrix production (Yamane et al., 2016) and SOD2, an anti-oxidant response gene (Mercado et al., 2019). We also checked for the putative effect of AAT on the expression of sterol regulatory element-binding transcription factor 1 (SREBF1), known as sterol regulatory element-binding protein 1 (SREBP-1) that induces adaptive responses to various stresses to maintain membrane lipid composition and ensure cell survival (Hagen et al., 2010). Both AAT proteins significantly induced expression of the *SREBF1* gene (**Figure 6**). This latter finding may in part explain AAT-induced decrease in *FLG2* expression, since *FLG2* is one of the genes that are downregulated after cholesterol depletion of keratinocytes (Mathay et al., 2011).

### Global Changes in the Transcriptome of EpiCS Treated With Human AAT

**Figure 7:**
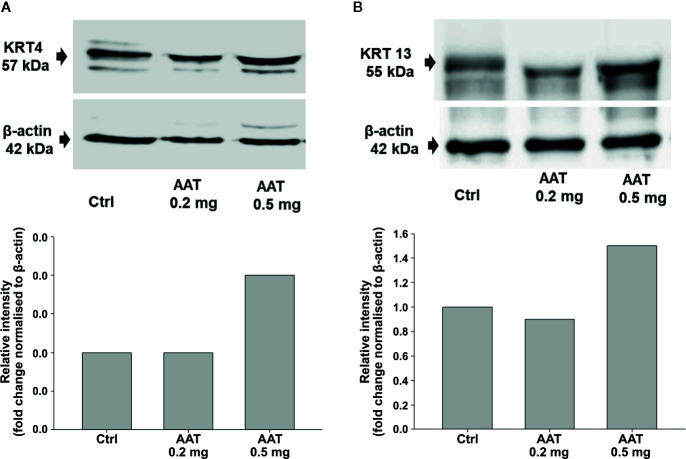
Effect of AAT on KRT 4 **(A)** and KRT 13 **(B)** proteins. EpiCS were cultured for 18 h with topically applied HBSS (control) or with 0.2 and 0.5 mg human AAT. Cell lysates were subjected to Western blot analysis using anti-KRT4 and anti-KRT13 antibody (1:500). β-actin was used as a loading control. Representative blots are shown. Bars show relative intensity of KRT4/13 bands from two independent experiments.

RNA-seq analysis was carried out by using total RNA prepared from epiCS treated for 18 h with topically added 0.2 mg AAT or HBSS. According to our results, AAT altered expression of the 873 genes with absolute GFOLD value (**Supplementary Table 1**). The AAT-regulated genes identified by RNA-seq were evaluated for functional biological processes and enrichment using the analysis tool from the PANTHER Classification System, which is maintained up to date with GO annotations (**Table 2**). The functional biological processes with the highest level of enrichment were retinoic acid biosynthesis and diterpenoid biosynthesis. Among the top AAT-upregulated genes were keratins and collagens. In line, RT-PCR analysis confirmed that topically applied 0.2 mg AAT induces expression of *KRT4* and *KRT13*, and *COL7A1* genes but has no effect on *VIM* expression (**Table 3**). According to the western blotting data, KRT4 and KRT13 proteins are already induced with a lower amount of only 0.5 mg AAT (**Figure 7**).

**Table 2:** Global changes in the transcriptome of epiCS treated with human AAT: GO enrichment analysis.

GO Term	Genes in Term	Genes Affected	Enrichment	P-value	FDR
retinoic acid biosynthetic process (GO:0002138)	9	5	13.79	1.29E-04	1.10E-02
diterpenoid biosynthetic process (GO:0016102)	10	5	12.41	1.87E-04	1.48E-02
cornification (GO:0070268)	113	30	6.59	2.69E-14	2.67E-11
intermediate filament organization (GO:0045109)	23	6	6.47	7.34E-04	4.80E-02²
peptide cross-linking (GO:0018149)	34	8	5.84	1.79E-04	1.43E-02
keratinization (GO:0031424)	226	49	5.38	2.49E-19	7.91E-16
keratinocyte differentiation (GO:0030216)	268	55	5.09	1.36E-20	7.20E-17
apoptotic mitochondrial changes (GO:0008637)	58	11	4.71	6.45E-05	6.04E-03
epidermal cell differentiation (GO:0009913)	313	57	4.52	3.67E-19	9.74E-16
intermediate filament cytoskeleton organization (GO:0045104)	51	9	4.38	4.72E-04	3.31E-02

**Table 3:** Gene expression analysis in epiCS treated with 0.2 mg AAT using RT-PCR.

Condition	Gene expression relative to housekeeping gene HPRT Mean and p-value							
*Repeats N = 6*	*COL7A1*		*KRT4*		*KRT13*		*VIM*	
**Control**	17.6 (3.2)		1.4 (0.5)		2.0 (0.7)		18.9 (0.9)	
**AAT**	30.1 (3.5)	p < 0.001	4.4 (1.3)	p < 0.001	5.7 (1.6)	p < 0.001	19.1 (1.1)	N.S.

N.S., not significant.

### Morphological Changes in EpiCS Cultured in the Presence of LPS/PGN Without or With Topical Application of AAT

**Figure 8:**
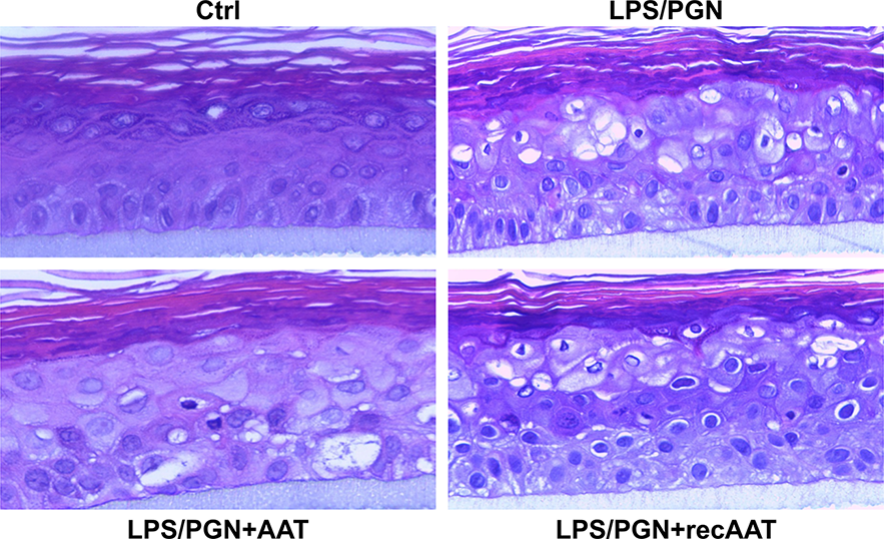
Typical images of the tissue architecture of the epiCS cultured for 18 h in the medium supplemented with 100 µg of LPS/PGN without or with topical application of 0.2 mg of human AAT or recAAT. The epidermis model was formalin-fixed, embedded in paraffin, cut, and stained with hematoxylin/eosin as described in *Materials and Methods*. Images were taken at 40× magnifications with the iRiSTM Digital Cell Imaging multicolor fluorescence system.

In this set of experiments, we cultured epiCS in medium containing a mixture of 100 µg/ml LPS/PGN. As illustrated in **Figure 8**, epiCS challenged with LPS/PGN mixtures displayed many small irregular-shaped holes in addition to the larger more rounded holes. Moreover, aggregation of high-density materials occurred at the rim of some of these structures. A closer visual analysis of the intercellular space between keratinocytes showed that many of them are not tightly connected. However, epiCS that were topically treated with 0.2 mg AAT or recAAT and cultured in LPS/PGN containing medium, showed a clear trend of less damage in the cell layers and more tight connection among keratinocytes (**Figure 8**).

### Effects of AAT on Filaggrin Protein Levels and Expression in EpiCS Activated With LPS/PGN

**Figure 9:**
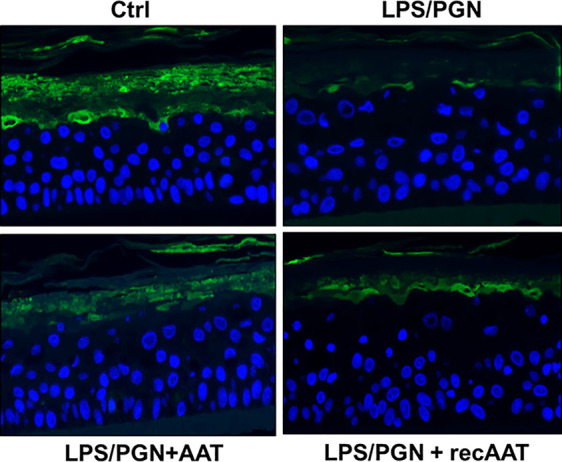
Immunofluorescence microscopy of filaggrin (FLG) in the upper layers of epiCS. The epiCS were cultured for 18 h in the medium supplemented with 100 µg of LPS/PGN without or with topical application of 0.2 mg of human AAT or ecAAT and processed as described in *Materials and Methods*. Sections were immunolabeled with mouse monoclonal IgG1 (kappa light chain) anti-filaggrin 1:500, (AKH1, sc66192c, Santa Cruz) and mounted by using a drop of ProLong™ Gold Antifade Mountant containing DAPI. Images were taken at 20× magnification using the iRiSTM Digital Cell Imaging multicolor fluorescence system (Logos Biosystems). Representative images show filaggrin (*in green*) and nucleus *(in blue)*. Scale bars: 20 µm (not shown).

The culture of epiCS in the medium supplemented with LPS-PGN mixture resulted in a strong down-regulation of the FLG protein on the outmost layer of epidermis. Under the same condition, topical application of 0.2 mg AAT or recAAT reduced this effect of LPS/PGN (**Figure 9**). As expected, *FLG2* expression was down-regulated 1.4-fold in epiCS cultured with LPS/PGN relative to controls [*FLG2* mRNA relative to housekeeping gene HPRT1, mean, n = 3 experiments: LPS/PGN 0.86 (0.04) *vs* control 1.3 (0.04), p < 0.001]. The application of 0.2 mg AAT did not influence *FLG2* expression in epiCs treated with LPS/PGN: [*FLG2* mRNA relative to housekeeping gene HPRT1, mean n = 3 experiments, LPS/PGN + AAT 0.85 (0.07) *vs* LPS/PGN 0.86 (0.04), n.s.].

### Effects of Topically Applied AAT on Chemokine/Cytokine Expression and Release in LPS/PGN Treated EpiCS

**Figure 10:**
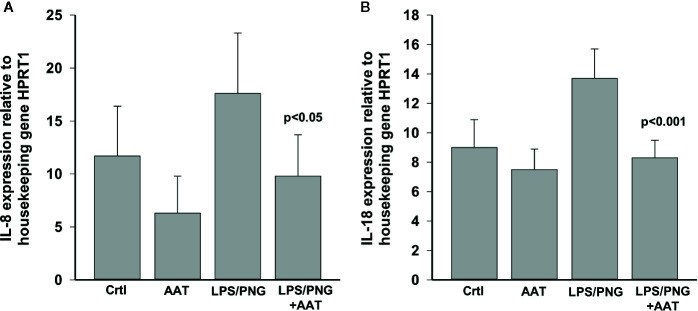
Effects of topically applied 0.2 mg human AAT and recAAT on *IL8* **(A)** and *IL18* **(B)** expression in epiCS cultured for 18 h in the presence of LPS/PGN. The expression of genes was determined by real-time PCR as described in *Materials and Methods*. HPRT1 was used as a housekeeping control. Bars represent mean from three independent experiments with two replicates for each condition. P value indicates significant differences between the values seen in LPS/PGN and LPS/PGN+AAT treated epiCS.

To evaluate the effect of LPS/PGN on pro-inflammatory cytokines, we measured IL-8 and total IL-18 levels. As shown in **Figure 10**, culture of epiCs in the presence of LPS/PGN led to enhanced expression of *IL8* and *IL18* mRNA by about 34% for both cytokines while topically applied 0.2 mg AAT inhibited this induction. Likewise, the levels of IL-8 in culture supernatants were lower by about 44% in LPS/PGN + AAT *versus* LPS/PGN treated epiCS [mean n = 3 independent experiments: LPS/PGN + AAT 238.8 (13.2) *vs* LPS/PGN 133.6 (22.1) pg/ml, p < 0.05]. Despite LPS/PGN-induced *IL18* mRNA, release of total IL-18 did not increase relative to controls [mean, n = 3 independent experiments: LPS/PGN 36.6 (13.4) *vs* controls 33.6 (10.3), respectively, n.s.]. As expected, epiCS cultured in the presence of LPS/PGN secreted more IL-6 (by about 70-fold) and TNFα (by about 14.2-fold) relative to controls. However, the topical application of AAT showed no effect on IL-6 and only slightly (by about 20%) lowered TNFα release (data not shown).

### RNA-Seq Data From LPS/PGN and LPS/PGN + AAT Treated EpiCS

According to the RNA-seq data, culture of epiCs in medium supplement with LPS/PGN resulted in altered expression of 4,008 genes with absolute GFOLD scores (**Supplementary Table 2**). Among 4,008 differentially expressed genes (DEGs), the number of down-regulated genes was 1,294, and the number of up-regulated genes was 2,714. We also performed GO-terms enrichment analysis (**Table 4**). Among top induced genes by LPS/PGN treatment were *IL8* and *MMP9* whereas *FLG2* was among down regulated genes.

**Table 4:** Biological processes these DEGs from LPS/PGN treated epiCS.

GO Term	Genes in Term	Genes Affected	Enrichment	P-value	FDR
antigen processing and presentation of exogenous peptide antigen via MHC class I, TAP-independent (GO:0002480)	9	8	4.84	0.00217	0.0473
COPII-coated vesicle cargo loading (GO:0090110)	13	10	4.19	0.00131	0.0322
establishment of skin barrier (GO:0061436)	24	18	4.08	2.15E-05	0.000876
regulation of water loss via skin (GO:0033561)	27	20	4.03	8.63E-06	0.000389
regulation of podosome assembly (GO:0071801)	15	11	3.99	0.000991	0.0257
negative regulation of cytokine production involved in inflammatory response (GO:1900016)	17	11	3.52	0.00204	0.045
protein insertion into ER membrane (GO:0045048)	22	14	3.46	0.000587	0.0164
cellular response to arsenic-containing substance (GO:0071243)	19	12	3.44	0.0015	0.0359
positive regulation of transcription from RNA polymerase II promoter involved in cellular response to chemical stimulus (GO:1901522)	23	13	3.08	0.00203	0.0449
IRE1-mediated unfolded protein response (GO:0036498)	55	31	3.07	2.99E-06	0.000152

**Figure 11:**
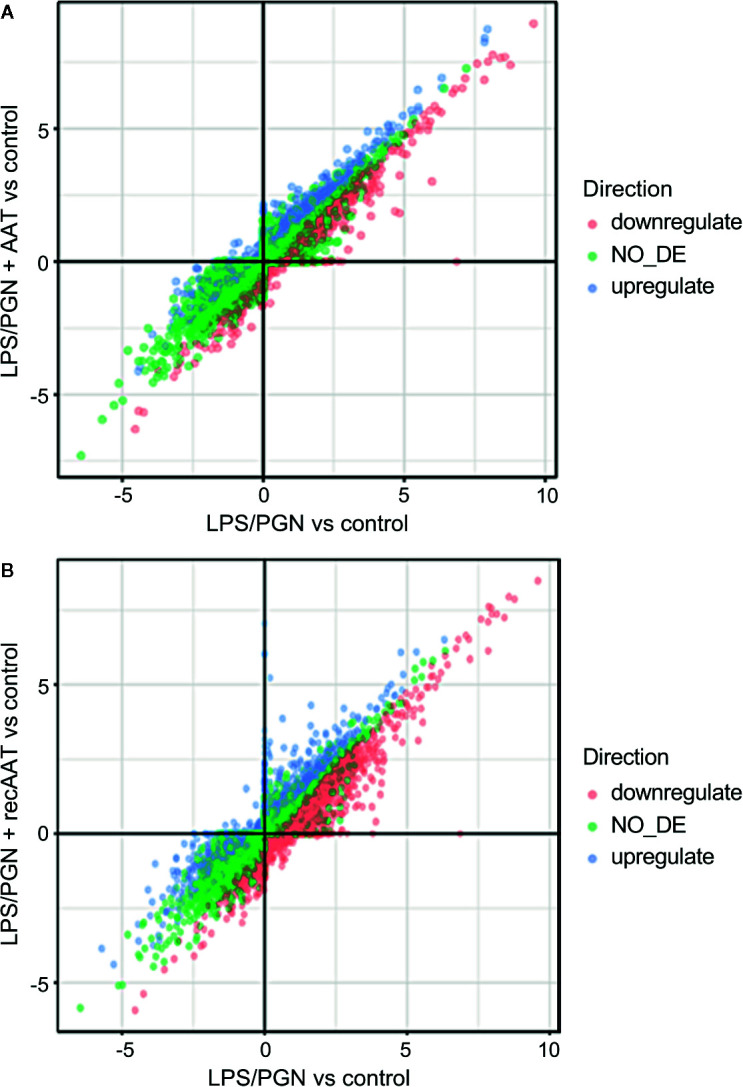
Comparison of two differential expression experiments: **(A)** LPS/PGN *vs* control and PLS/PGN + AAT *vs* Control; **(B)** LPS/PGN *vs* control and PLS/PGN+recAAT *vs* Control. Each axis represents the GFOLD score for the respective DE comparison. Color is used to depict which genes were up, down, or not regulated in the comparison of LPS/PGN + AAT (or LPS/PGN + recAAT) *vs* LPS/PGN.

Remarkably, under these experimental conditions, topical application of human plasma or recAAT affected only 44 and 45 genes, respectively, with absolute GFOLD. No GO terms were significant for these genes. As illustrated in **Figures 11A, B**, both AAT proteins showed similar pattern in the regulation of LPS/PGN-affected genes. Under these experimental conditions, most of the genes remained unaffected by AAT.

## Discussion

Skin is the largest organ of the body and an important site for drug delivery, especially for the treatment of primary skin conditions, autoimmune diseases and infections (Prow et al., 2011). However, drug delivery approaches via the skin are challenging because of the limited permeability of most substances through the stratum corneum, the outermost layer (lipid matrix) of the epidermis. According to Rule of Five proposed by Lipinski in 1997 (Lipinski et al., 2001), protein drugs, which are large (average weight about 53 kDa) and hydrophilic, are not suitable for efficient transdermal delivery. Indeed, passive diffusion of larger proteins is poor and requires time, and thus often considered as insignificant (Munch et al., 2017). On the other hand, passive diffusion through the skin is an easy method to apply substances to large surface areas with minimal dermal damage and therefore remains of interest to explore further.

In this study, our main aim was to investigate a passive diffusion of AAT protein (about 52 kDa, dependent on the glycosylation pattern) through 3D epidermis inserts, a reconstructed skin equivalent. This model consists of human keratinocytes grown on underlying microporous membranes ensuring contact to the cell culture media, and is widely used instead of animals for testing the efficacy and toxicity of various pharmaceutical products, as well as raw materials (Mertsching et al., 2008); (Hoffmann et al., 2005). Because previous pilot studies showed inconsistent data with topical application of human and recombinant AAT proteins (Wachter and Lezdey, 1992; Mazereeuw-Hautier et al., 2006), we used two AAT proteins, one purified from human plasma and another recombinantly produced by CHO cells. Both proteins were highly purified and had a similar glycosylation pattern and biological activity. Herein, we used 0.2 and 0.5 mg of AAT based on the knowledge that these concentrations express *in vivo* biological activities (Hubbard and Crystal, 1990; Aldonyte et al., 2004).

A study by Lockett et al. found that regardless of whether AAT was added to the apical or the basolateral side of the endothelium, the protein could be detected intracellularly and was secreted across the trans-well membrane (Lockett et al., 2014). Likewise, our results clearly show that topical application the same volume of human or recombinant AAT results in a free diffusion of proteins across the epiCS in a concentration and time-dependent manner, and that AAT protein reaches the medium compartment. Moreover, we found that AAT passively diffuses all layers of epiCS without causing obvious morphological alterations and can be detected in cell cytosol. Because the topical application of 0.2 mg of AAT showed good passive diffusion within 18 h, this condition was selected for our further experiments.

In subsequent experiments, we examined the tolerability of epiCs to AAT protein. For comparison, the Hank’s balanced solution (in which AAT proteins were prepared) was applied under equal conditions. Keratinocytes are main epidermal cells, which can produce many cytokines in response to stimuli. Here we selected IL-18, IL-1α, IL-8, and IL-6 as immunological markers to evaluate whether topical application of AAT has activating or irritating effects on epiCS. Pro-inflammatory cytokine, IL-18, is constitutively expressed and released by human keratinocytes *in vitro* (Mee et al., 2000) but can be activated during various inflammatory conditions. In response to physical or chemical stresses, keratinocytes produce and release other inflammatory cytokines like IL-1α and the chemotactic cytokine IL-8. IL-1α has an effect on epidermal differentiation and is highly relevant in a response to mechanical injury of the skin. Deregulated expression of IL-1α promotes an inflammatory skin phenotype (Hanel et al., 2013) and stimulates the release of secondary mediators, such as IL-8, which promote the migration of innate immune cells and the overall initiation of cutaneous inflammation (Frankova et al., 2018). We did not observe time-dependent differences regarding the IL-1α and total IL-18 release from epiCS treated with human or recombinant AAT, or with HBSS. Likewise, AAT did not induce IL-8 release from epiCS cultured for 18 h. In fact, epiCS treated with human AAT showed a slight but significant reduction in IL-8 release as compared controls. Recent studies have shown that chemical and mechanical stimuli can increase the IL-6 production by keratinocytes (Oh et al., 2019). Again, topically applied AAT did not induce IL-6 release. We also measured TNFα; however, here levels were below detection limits.

Taken together, these above results support the notion that a topical application of AAT does not induce stress, which could induce inflammatory cytokine release. Likewise, the expression of highly inducible genes in response to pro-inflammatory insults, such as *COX2*, *SOD2* and *TGFB1* genes (Wu, 1996; Yamane et al., 2016; Mercado et al., 2019), was not stimulated by the topical application of AAT proteins. To the contrary, human AAT reduced *COX2* gene expression relative to controls or epiCS treated with recombinant AAT. Some studies suggest that COX-2 inhibition prevents skin inflammation and aging (Lee et al., 2012). On the other hand, the expression of *SREBF1* gene increased significantly in epiCS treated with human or recombinant AAT. Using genome-wide expression analysis, researchers identified *SREBF1* as a modulator of genes that drive fatty acid and cholesterol synthesis to maintain lipid homeostasis (Ayala-Sumuano et al., 2011). SREBF1-mediated lipid synthesis seems to be important to maintain integrity and functions of lipid raft membrane (Lee et al., 2018b). Presently we are not able to answer definitely, if AAT-induced *SREBF1* expression is directly linked with the passive diffusion of AAT *via* lipid-rafts. Mathay and co-authors have shown that disruption of lipid rafts results in the downregulation of filaggrin-2 (*FLG2*) expression (Mathay et al., 2011). These authors found that *FLG2* is specifically downregulated after cholesterol depletion of keratinocytes. In our experimental model, topically applied AAT significantly reduced *FLG2* expression. Thus, the observed parallel induction of *SREBF1* and reduction of *FLG2* expression allow speculating that there is a link between the transdermal trafficking of AAT and the integrity of the lipid raft microdomains. We and other investigators have previously shown that AAT enters cells *via* lipid rafts (Bergin et al., 2010; Subramaniyam et al., 2010). As previously pointed out, the lipids of the stratum corneum are considered the major determinant of drug absorption by the skin (Kalia and Guy, 2001; Alexander et al., 2012; Desai et al., 2013; Frasch and Barbero, 2013; Notman and Anwar, 2013). The properties of AAT to interact with hydrophobic substances (e.g. fatty acids and lipoproteins) and its ability to enter cells *via* lipid rafts probably are essential for the passive diffusion of AAT through the layers of epiCS.

Some investigations have shown a link between activation of COX-2 signaling and down-regulation of FLG in the skin (Karuppagounder et al., 2015). However, AAT showed no effect on COX-2 expression and did not induce pro-inflammatory cytokine production. Intriguingly, although AAT lowered *FLG* gene expression it had no significant effect on FLG protein levels in epiCS. Therefore, it is important to keep in mind that the regulation of FLG expression by AAT might be not necessarily restricted to changes in transcription; post‐transcriptional changes to mRNA stability may also be involved. FLG is a major component of epidermal cells (Brown and Mclean, 2012), which modulates epidermal homeostasis (Dang et al., 2015). Up to date, the modulation of FLG levels by AAT has not been studied. Therefore, detailed investigations on the AAT transdermal penetration and pathways involved will be part of our continuing research projects.

Yet, our RNA-seq data show that topical application of 0.2 mg AAT to epiCS affects the expression of 873 genes selected with a GFOLD value. Most of these genes seem to be involved in the retinoic acid biosynthetic process, intermediate filament organization, and keratinocyte differentiation. Among affected genes in epiCS treated with AAT, were significant upregulated keratins, like KRT4 and KRT13, and collagens, specifically collagen type VII alpha 1 Chain (COL7A1). A previous study proposed that the upregulation of KRT4/KRT13 in human keratinocytes is mainly mediated *via* retinoic acid receptors (Virtanen et al., 2010). According to our RNA-seq data, most of the regulated genes by AAT were related to retinoic acid biosynthetic process. These pilot findings open a new avenue for investigation a link between AAT and expression of KRT4 and KRT13, often paired in skin models. Moreover, AAT upregulates *COL7A1* expression that is not only vital for maintaining skin integrity, but is also a critical player in wound closure (Nystrom et al., 2013). This latter finding supports the previously suggested role of AAT in wound healing (Congote et al., 2008; Lewis, 2012).

Finally, we asked whether topically applied AAT has any protective effects on inflamed epiCS. To this end, we supplemented our epiCS culture medium with a LPS/PGN mixture with or without topical application of 0.2 mg of AAT and incubated the cultures for 18 h. The reason for employing a LPS/PGN combination (two bacterial glycoconjugates: LPS, from the outer membrane of gram-negative bacteria and PGN, from the cell walls of both gram-positive and gram-negative bacteria) was to induce a broad pro-inflammatory response of our epidermis model. The culture of epiCs in the medium supplemented with LPS/PGN resulted in morphological alterations and in expression changes of 4,008 genes, many of which were related to establishment of skin barrier, regulation of water loss *via* skin and cytokine production. Notably, RNA-seq data revealed that in our LPS/PGN-treated skin model topical application of AAT or recombinant AAT affected only 44 and 45 genes, respectively, and showed no influence on GO pathways. However, histological and biological examinations of specimens treated with AAT revealed that, generally speaking, AAT exhibits protective properties. For example, AAT diminished the property of LPS/PGN to decrease FLG protein levels and inhibited induced IL-8 expression and release. In line with previously published data (Companjen et al., 2000), we found that LPS/PGN treatment does not significantly affect the release of IL-18. Nevertheless, topical application of AAT significantly lowered IL-18 expression in LPS/PGN-stimulated epiCS.

In conclusion, our results support historical data and provide new evidence that topical application of AAT might be possible due to its ability to diffuse passively via epidermis. Why can AAT penetrate into epidermis? The intercellular lipid matrix forms a continuous pathway from the skin surface to the viable skin tissues creating the path of entry for many substances. Therefore, the nature of the extracellular lipid matrix plays a critical role for the permeability of human skin. Human skin lipids consist of free fatty acids, ceramides, and cholesterol in an approximately equimolar ratio (De Luca and Valacchi, 2010). A large conformational flexibility and variability in hydrophobicity among AAT protein conformers (Patschull et al., 2012), together with findings that AAT interacts with cholesterol, fatty acids, and lipid rafts allowed assuming that AAT may interact with lipid matrix and penetrate lipid layers of skin surface. On the other hand, patients suffering from inflammatory skin disorders, like atopic dermatitis, psoriasis, or Netherton syndrome, possess the impaired skin barrier function that may contribute to increased drug absorption. Previous studies have shown that the degree of epidermal barrier disruption correlates with degree of skin inflammation (Sator et al., 2003), and that the periodicity and general appearance of the lipid matrix is altered in inflamed skin (Madison, 2003). These changes in the skin barrier may benefit topical entry of AAT. Topical preparations of AAT may either dampen the epidermal response to barrier disruption or enhance barrier recovery. To confirm these predictions, would require the examination of tissue-specific changes in both animal models and human patient samples.

The limitation of this epiCS model is the lack of a dermal part although the fibroblasts very seldom play a metabolically active role—they are mostly active in wound healing processes (Chang et al., 2002). Besides, the stratum corneum on any reconstructed skin model is likely more permeable than actual intact human skin (Neupane et al., 2020). Novel methods recently became available which are capable of delivering larger substances (peptides and proteins) through the skin (Asselineau et al., 1990; Prow et al., 2011). The various approaches available include iontophoresis, which consists on the application of low intensity electric current (Gelfuso et al., 2008), sonophoresis with application of low-frequency ultrasound (Polat et al., 2010), and microneedles, which are minimal invasive needle-like micro projections (Moffatt et al., 2017). All these strategies are extensively studied to facilitate transdermal drug delivery (Moffatt et al., 2017; Munch et al., 2017; Lee et al., 2018a), and likely to be useful for the delivery of AAT, as well. Therefore, our findings obtained by using skin surrogates encourage using more advanced models for detailed investigations of the penetration of AAT *via* skin layers and the consequences of its application, both locally and systemically. Different solvents need to be evaluated for the optimal delivery of topical AAT. For this animal models *in vivo* can be used. If confirmed, transepidermal therapy with AAT, as non-steroid immunomodulatory biopharmaceutical, would be of great value for patients who develop skin disorders.

## Data Availability Statement

The data generated from this article can be found in NCBI using the accession number GSE150946.

## Author Contributions

ST: experiments with epiCs, immunostainings, western blot analysis. BM-D and GG-M: RNA-seq analysis. BL and DD: statistical analysis and data presentation. EK: confocal microscopy. DJ: immunohistochemistry. FW and MW: recombinant protein synthesis and purification. FJ: three dimensional (3D), fully differentiated human epidermis equivalent preparation. TW: project administration, resources. SJ: conceptualization, supervision, writing—original draft preparation. All authors performed writing—reviewing and editing of the manuscript.

## Funding

This study was supported partly by research funds from ExcellGene SA and by the National Science Center, Poland (grant 2015/17/B/NZ5/01370) and ALTA award for ST (fond number: 19400569).

## Conflict of Interest

Authors FW and MW are employed by ExcellGene SA, which provided the recombinant AAT. They also discussed with the lead author (SJ) the approaches for studying the use of AAT during experiments. They declare that their contribution to the research shall not be construed as a potential conflict of interest.

The remaining authors declare that the research was conducted in the absence of any commercial or financial relationships to the company that could be construed as a potential conflict of interest.

The authors declare that this study received funding from ExcellGene SA. The funder had the following involvement with the study: ExcellGene provided purified recombinant AAT protein, which we used as a comparison to plasma AAT, and supported financially the purchase certain skin model kits. The funders had no role in study design, data collection and analysis, decision to publish, or preparation of the manuscript.

## Supplementary Material

Supplementary figure 1 Representative images of hematoxylin/eosin staining from 3D reconstructed human epidermis culture incubated for 18 h without or with topical application of 0.5 mg of human AAT. The epidermis model was formalin-fixed, embedded in paraffin, cut, and stained with hematoxylin/eosin. Images were taken at 40× magnifications with the iRiSTM Digital Cell Imaging multicolor fluorescence system. We did not observe significant morphological differences between controls and AAT treated epiCS.

Supplementary figure 2 Free diffusion of topically applied 0.2 mg **(A)** and 0.5 mg **(B)** AAT after 18 h. Staining for AAT (brown color) indicated by arrows. Images were taken at 100× magnifications using Leica DM750 microscope equipped with Leica ICC50 HD camera.

Supplementary figure 3 Immunofluorescence microscopy analysis of 0.2 mg AAT added basolaterally **(A)** and topically **(B)** to epiCS for 18 h. Specimens were stained with polyclonal rabbit anti-human AAT antibody (Mazereeuw-Hautier et al.) and DAPI (red). Images were acquired using Olympus FluorView 1000 scanning confocal microscope equipped with a 60× oil immersion objective. Scale bar: 10 μm (not shown).

Supplementary figure 4 Effects of human AAT and recAAT on IL-8 and total IL-18 release. EpiCS were incubated for 18 h in either topically applied buffer alone or human AAT or recAAT (2 mg/ml). ELISA determined released levels of IL-8 **(A)** and total IL-18 **(B)**. Bars represent mean from three individual epiCs. P value indicates significant differences compared with the values seen in controls.
