# Supplementary figures and images for: The Delivery of α1-Antitrypsin Therapy Through Transepidermal Route: Worthwhile to Explore
Source: Front Pharmacol. 2020 Jul 3;11:983. doi: 10.3389/fphar.2020.00983 (PMC7348051; doi:10.3389/fphar.2020.00983)

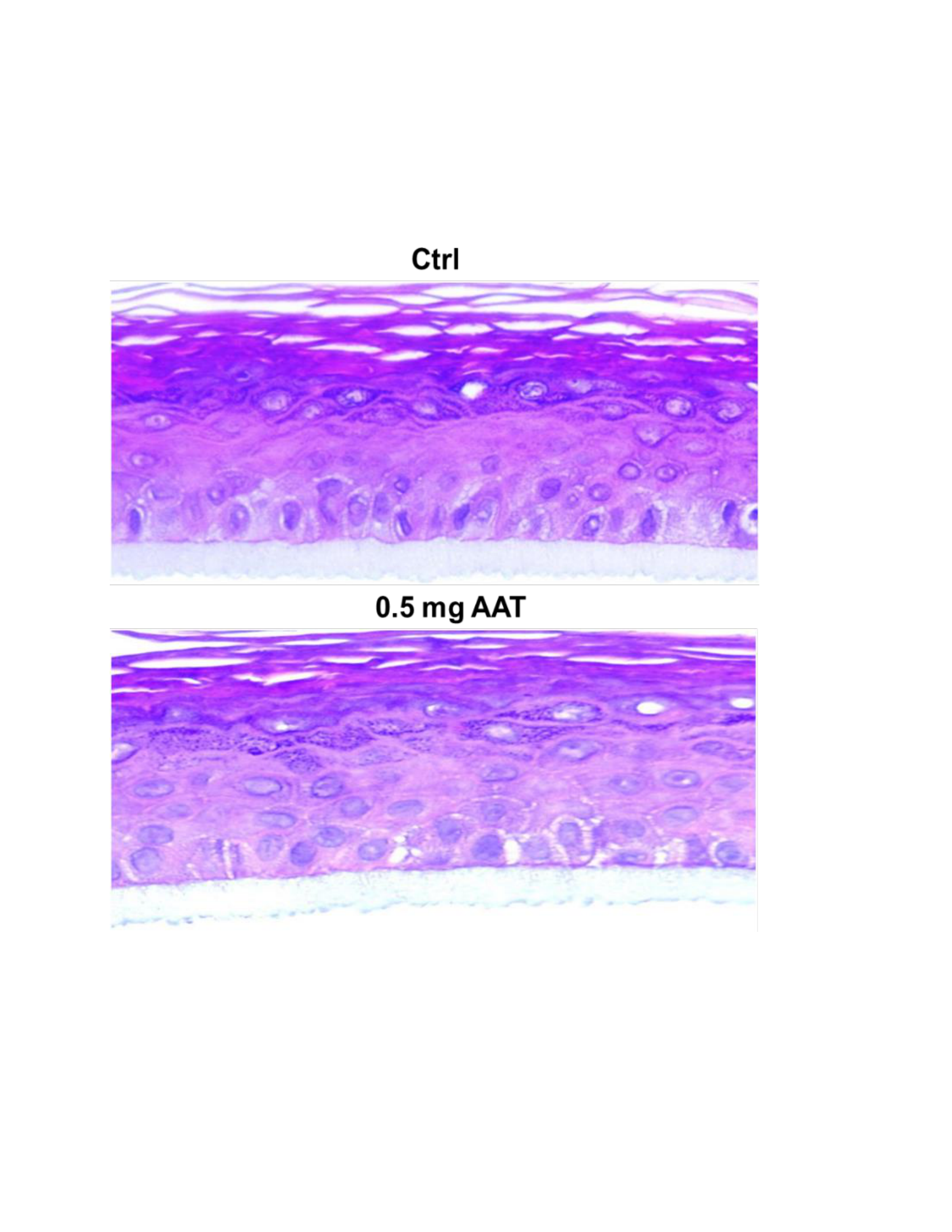

Supplement: Supplementary figure 1 — Representative images of hematoxylin/eosin staining from 3D reconstructed human epidermis culture incubated for 18 h without or with topical application of 0.5 mg of human AAT. The epidermis model was formalin-fixed, embedded in paraffin, cut, and stained with hematoxylin/eosin. Images were taken at 40× magnifications with the iRiSTM Digital Cell Imaging multicolor fluorescence system. We did not observe significant morphological differences between controls and AAT treated epiCS. [file Image_1.tiff]

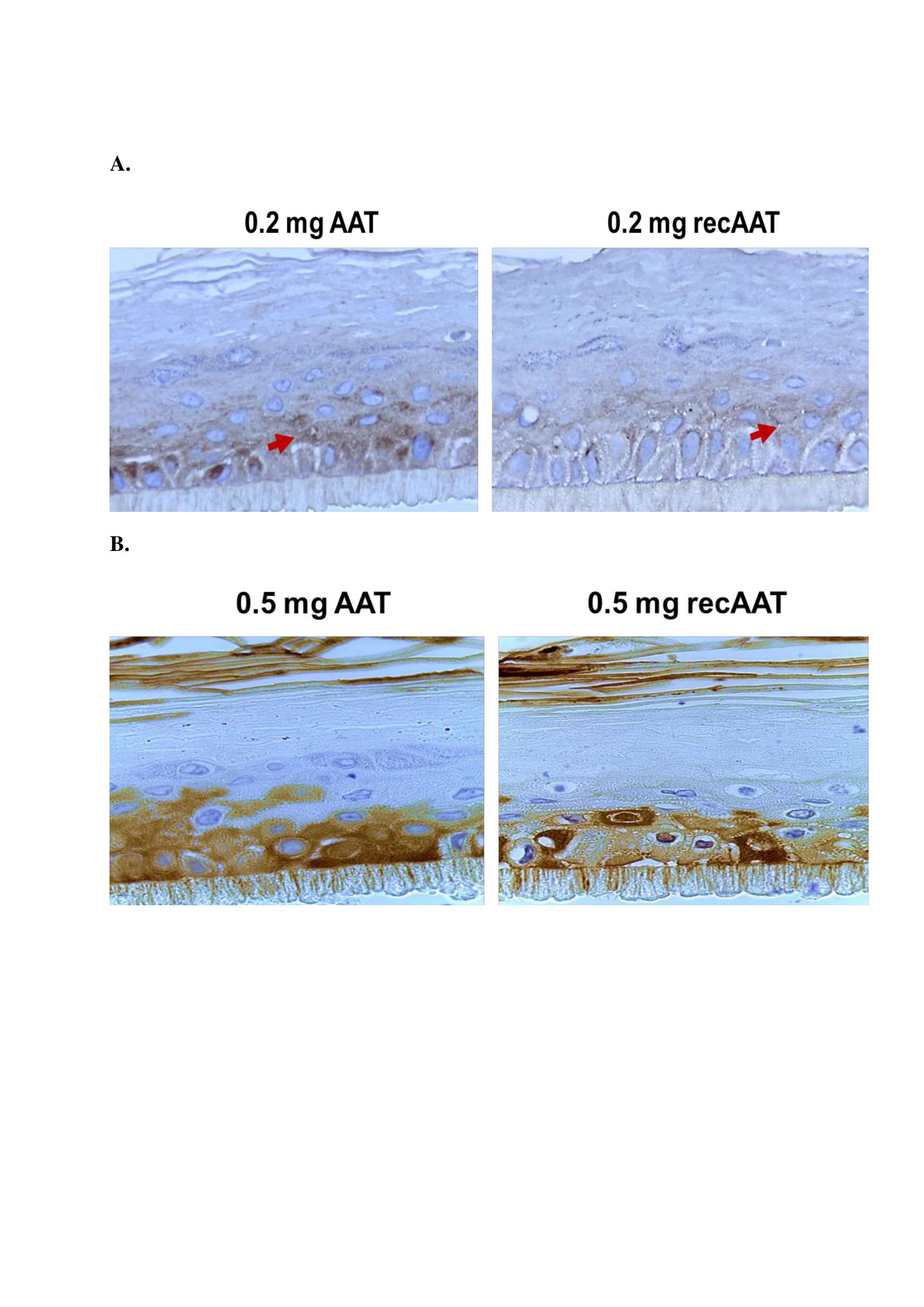

Supplement: Supplementary figure 2 — Free diffusion of topically applied 0.2 mg (A) and 0.5 mg (B) AAT after 18 h. Staining for AAT (brown color) indicated by arrows. Images were taken at 100× magnifications using Leica DM750 microscope equipped with Leica ICC50 HD camera. [file Image_2.tiff]

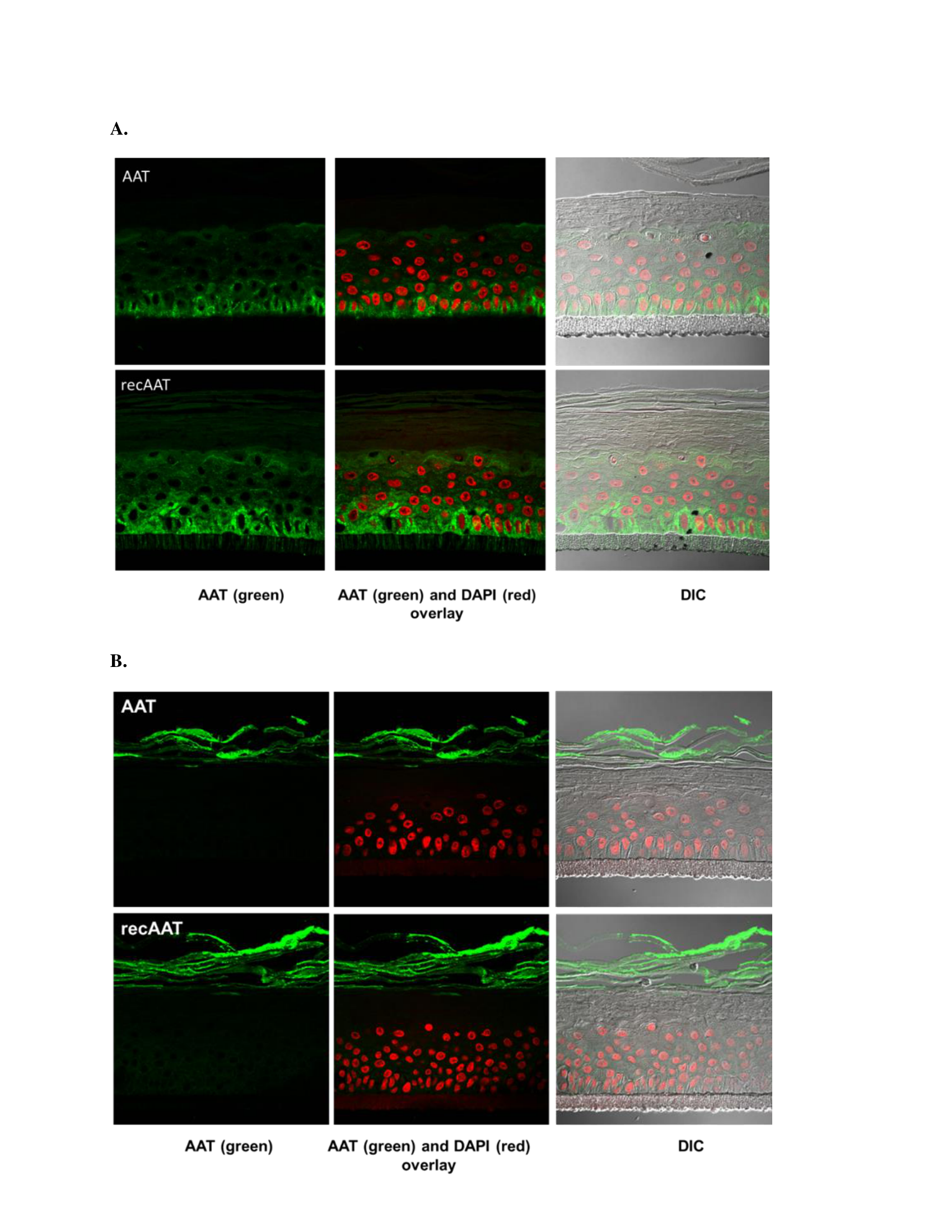

Supplement: Supplementary figure 3 — Immunofluorescence microscopy analysis of 0.2 mg AAT added basolaterally (A) and topically (B) to epiCS for 18 h. Specimens were stained with polyclonal rabbit anti-human AAT antibody (Mazereeuw-Hautier et al.) and DAPI (red). Images were acquired using Olympus FluorView 1000 scanning confocal microscope equipped with a 60× oil immersion objective. Scale bar: 10 μm (not shown). [file Image_3.tiff]
